# Meniscal body extrusion and cartilage coverage in middle-aged and elderly without radiographic knee osteoarthritis

**DOI:** 10.1007/s00330-018-5741-3

**Published:** 2018-10-02

**Authors:** Fredrik Svensson, David T Felson, Fan Zhang, Ali Guermazi, Frank W Roemer, Jingbo Niu, Piran Aliabadi, Tuhina Neogi, Martin Englund

**Affiliations:** 1 Faculty of Medicine, Department of Clinical Sciences Lund, Orthopedics, Clinical Epidemiology Unit, Lund University, Lund, Sweden; 2 Clinical Epidemiology Research & Training Unit, Boston University School of Medicine, Boston, MA USA; 3 Department of Radiology, Boston University School of Medicine, Boston, MA USA; 4 Department of Radiology, University of Erlangen-Nuremberg, Erlangen, Germany; 5 Brigham and Women’s Hospital, Harvard Medical School, Boston, MA USA

**Keywords:** Knee, Meniscus, Magnetic resonance imaging, Osteoarthritis, Coverage

## Abstract

**Objectives:**

To determine meniscal extrusion and cartilage coverage on magnetic resonance (MR) images and factors associated with these parameters in knees of middle-aged and elderly persons free from radiographic tibiofemoral osteoarthritis (OA).

**Methods:**

Seven hundred eighteen persons, free of radiographic tibiofemoral OA, aged 50–90 years from Framingham, MA, USA, were included. We measured meniscal extrusion on 1.5 T MRI of both knees to evaluate both medial and lateral meniscal body extrusion and cartilage coverage. We also determined meniscal morphology and structural integrity. The multivariable association with age, body mass index (BMI), and ipsilateral meniscal damage was also evaluated.

**Results:**

The mean meniscal body extrusion medially was 2.7 mm and laterally 1.8 mm. The tibial cartilage coverage was about 30% of ipsilateral cartilage surface (both compartments). The presence of ipsilateral meniscal damage was associated with more extrusion in only the medial compartment, 1.0 mm in men and 0.6 mm in women, and less cartilage coverage proportion, -5.5% in men and -4.6% in women.

**Conclusions:**

Mean medial meniscal body extrusion in middle-aged or older persons without radiographic tibiofemoral OA approximates the commonly used cutoff (3 mm) to denote pathological extrusion. Medial meniscal damage is a factor associated with medial meniscal body extrusion and less cartilage coverage.

**Key Points:**

*• Medial meniscal extrusion in middle-aged/older persons without OA is around 3 mm.*

*• Lateral meniscal extrusion in middle-aged/older persons without OA is around 2 mm.*

*• Meniscal damage is associated with medial meniscal extrusion and less cartilage coverage.*

**Electronic supplementary material:**

The online version of this article (10.1007/s00330-018-5741-3) contains supplementary material, which is available to authorized users.

## Introduction

The menisci of the knee are two fibrocartilaginous discs located on the medial and lateral sides of the knee joint. Their primary purpose is to distribute loads over a broad area of the articular cartilage between femur and tibia [1–3]. Meniscus extrusion, i.e., when the peripheral border of the meniscus is substantially located *outside* the joint margin, has been reported to be associated with meniscal degeneration, meniscus tears, and the presence and progression of osteoarthritis (OA) [4–22]. However, there is very limited information of extent of meniscus extrusion and cartilage coverage of the meniscus of middle-aged and elderly free of tibiofemoral OA, i.e., not selected on the basis of attending a clinic as a patient, or having knee symptoms, or having risk factors for OA [23]. This information is needed to better understand what is “normal.” Further, there is still quite limited information on common demographic factors associated with meniscus body extrusion as, e.g., gender [16, 24–26]. Therefore, in the present original report, we asked the following questions: (1) what is the meniscal body extrusion and coverage in a sample representative of the general population of middle-aged and elderly without radiographic tibiofemoral OA? What are the potential associations with age, body mass index (BMI), and meniscus damage in men and women, respectively? For this purpose, we used a comprehensive cohort of over 700 persons representative of the general population from Framingham, USA, with knees considered radiographically normal.

## Material and methods

We used data from the well-characterized Framingham Community cohort [14, 27–29]. As detailed in prior work, the cohort consists of a random sample of 1039 persons from Framingham, MA, USA. The inclusion criteria and sampling have been detailed in a prior report [14]. In brief, the subjects were aged 50–90 years and were drawn from census tract data and random-digit telephone dialing. They were ambulatory and the selection was *not* made on the basis of knee or other joint problems. All subjects’ height and weight were measured. We obtained posterioanterior knee x-rays in weight-bearing using a fixed-flexion protocol. One musculoskeletal radiologist (PA), who was unaware of the MRI findings and clinical data, scored the radiographs according to the Kellgren and Lawrence (KL) scale (intraobserver kappa, 0.83) [14, 30]. In our study, we included only persons with KL grade 0 in *both* knees and with readable knee magnetic resonance (MR) images for at least *one* knee resulting in a sample of 718 persons.

### Knee MR imaging

MRI scans of 712 right knees and 674 left knees were obtained using a 1.5-T scanner (Siemens, Erlangen, Germany) with a phased array knee coil. We used three pulse sequences to assess meniscus position and integrity; sagittal and coronal fat-suppressed proton-density-weighted turbo spin-echo (repetition time 3610 ms, echo time 40 ms, 3.5-mm slice thickness, 0-mm interslice gap, echo spacing 13.2 ms, turbo factor 7, field of view 140 mm, matrix 256 × 256); and sagittal T1-weighted spin-echo (repetition time 475 ms, echo time 24 ms, 3.5-mm slice thickness, 0-mm interslice gap, field of view 140, matrix 256 × 256).

**Fig. 1:**
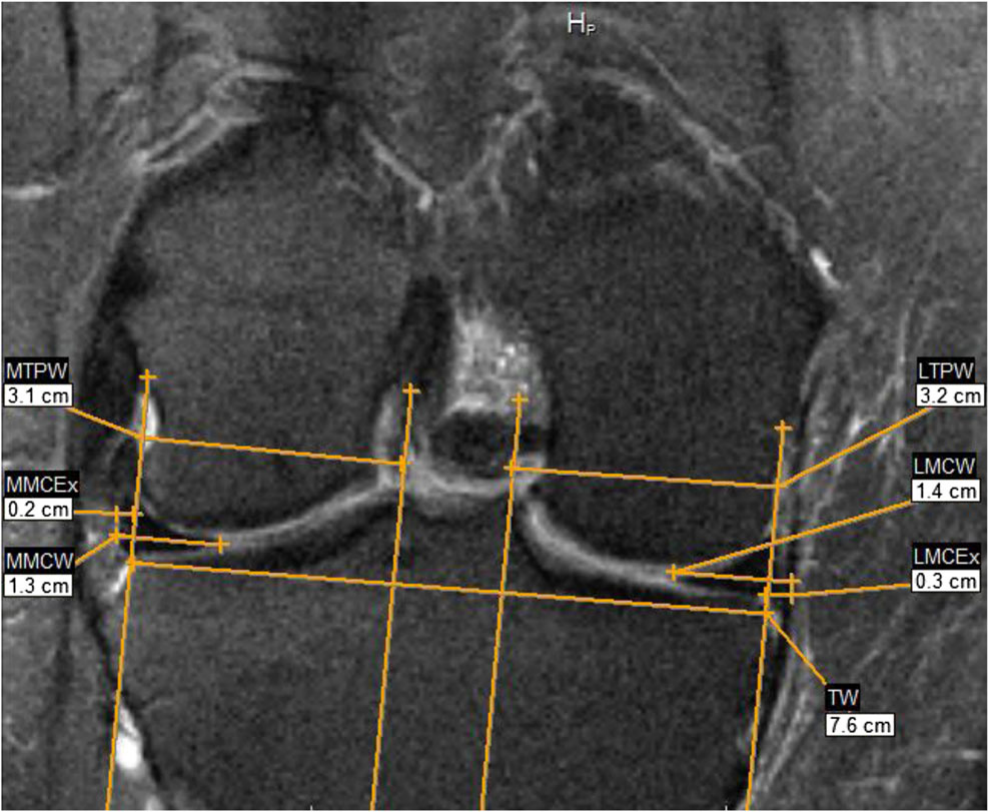
Example MR Image illustrating the measures. *TW* tibial width, *MTPW* Medial tibia plateau width, *LTPW* lateral tibia plateau width, *MMCW* medial meniscal coronal width, *LMCW* lateral meniscal coronal width, *MMCEx* medial meniscal coronal extrusion, *LMCEx* lateral meniscal coronal extrusion

Using the coronal images, one observer (FS, an orthopedic surgeon with more than 5 years of clinical experience incl. MRI) measured meniscal body extrusion to the nearest millimeter (mm) in both the medial and the lateral compartments of all knees where knee MRI was eligible for measurement of meniscus position. A subset of 30 knees was re-measured by the same observer as well as by a second reader (FZ, also an orthopedic surgeon with more than 5 years of clinical experience incl. MRI). The measurements were determined on the mid-coronal slice, where the medial tibia spine was of maximal area. When it was too difficult to distinguish the maximal spine area between two or more slices, the slice with the maximal tibia width was used. The point of reference for extrusion was the tibia plateau osteochondral junction at the joint margin excluding osteophytes [8, 12]. For the measurements, a reference line was drawn between the medial and lateral osteochondral junctions, defined as the tibia width. In 90° angle from this line, four gridlines were drawn. Then parallel to the tibia width the medial and lateral tibia plateau, the medial and lateral meniscal coronal width and the medial and lateral coronal meniscal extrusion were measured (Fig. 1). We excluded all subjects where the MR image was unreadable, or where the medial or lateral meniscal body was completely missing, i.e., no measure of meniscal extrusion could be obtained. We used Merge eFilm software 3.4 and made all the measurement to the closest mm.

As described in an earlier study of the Framingham cohort, a second reader (ME) (3-year clinical background in orthopedics incl. MRI) determined meniscus integrity (meniscus tear, maceration/destruction, and/or prior resection) on MR images of right knees [14]. All readers were blinded to the other readings and to clinical data.

### Statistics

We assessed the intra- (reader FS) and inter-observer reliability (readers FS and FZ) for all MRI measurements using the intra-class correlation coefficient (ICC).

We evaluated the distribution of the medial and lateral meniscal body extrusion in men and women, respectively. We also evaluated the meniscal body coverage, both the absolute measure in millimeter, but also defined as the proportion of the *width* of the mid-coronal ipsilateral tibia plateau covered by meniscus. Additionally, we presented means and standard deviations of meniscal body extrusion and coverage stratified by age (50–59, 60–69, and 70–90 years), body mass index (BMI) (< 25.0, 25.0–29.9, or 30.0+) or meniscus damage status (i.e., the presence or absence of either tear, maceration/destruction, and/or prior resection) as supplementary Web appendices.

Further, using a multivariable linear regression model with robust standard errors, we estimated the association of age (continuous), BMI (continuous), and meniscus damage (yes/no) with medial and lateral meniscal body extrusion and coverage, in men and women, adjusting for the tibia width to take the size of the knees into account. This multivariable analysis was only performed for right knees due to no data on meniscus integrity for left knees. For the statistical analysis, we used the Stata version 13.

## Results

### Study cohort characteristics

The mean (SD) age of our study sample was 61.0 (8.0) years and 55.3% were women. The mean BMI was 27.8 (Table 1).

**Table 1:** Descriptive statistics of the study sample from the general population of Framingham, MA, all knees were Kellgren and Lawrence grade 0

Characteristics	*n* = 718
Age, mean (SD) years	61.0 (8.0)
Sex, *n* (%)	
Men	321 (44.7)
Women	397 (55.3)
Body mass index, mean (SD) (kg/m^2^)	27.8 (5.1)
Meniscal damage in the right knee, *n* (%)*	165 (23.0)
Knee MRI eligible for measurement of meniscus position, *n* (%)	
Right knee	712 (99)
Left knee	674 (94)

*Only right knees have been read for meniscal integrity

The intra-reader reliability for FS for the medial meniscal extrusion was ICC = 0.89 (95% confidence interval [CI] 0.75, 0.96) and for the lateral meniscal extrusion ICC = 0.86 (95% CI 0.68, 0.94). The inter-rater reliability for FS and FZ for the medial meniscal extrusion was ICC = 0.69 (95% CI 0.43, 0.84) and for the lateral meniscal extrusion ICC = 0.68 (95% CI 0.42, 0.83).

### Meniscal body extrusion

The absolute values for meniscal body extrusion were similar in both men and women, with the mean 2.7 mm of extrusion in the medial compartment, and 1.8 mm in the lateral compartment for both sexes combined (Table 2, Fig. 1). The proportion of knees with medial meniscal body extrusion of 5 mm or more was 7% in men and 2.5% in women. The corresponding proportions for the lateral compartment were 1.4% and 0.5%. In knees *without* medial meniscal damage, the medial meniscal body extrusion was 2.4 mm for medial compartment (Web appendix 1).

**Table 2:** Mean absolute measure in mm (SD) of medial and lateral meniscal body extrusion for middle-aged and elderly persons with Kellgren and Lawrence grade 0 in both knees

	Right knee		Left knee	
	Medial	Lateral	Medial	Lateral
Men	2.7 (1.3)	2.1 (1.2)	2.9 (1.2)	1.6 (1.2)
Women	2.5 (1.1)	1.9 (1.2)	2.7 (1.1)	1.5 (1.0)
All	2.6 (1.2)	2.0 (1.2)	2.8 (1.1)	1.5 (1.1)

### Absolute meniscal coverage and coverage proportion

**Fig. 2:**
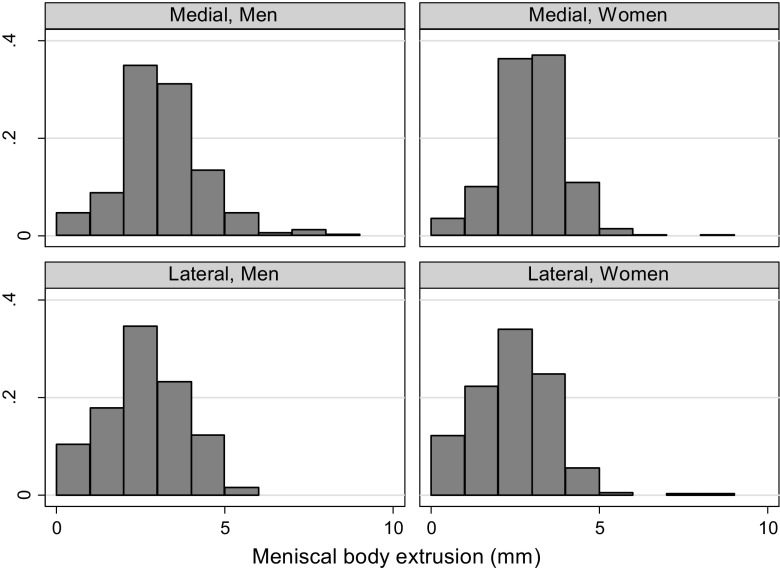
The distribution of the meniscal body extrusion in middle-aged and elderly persons with Kellgren and Lawrence grade 0 knees, stratified by sex and compartment

**Fig. 3:**
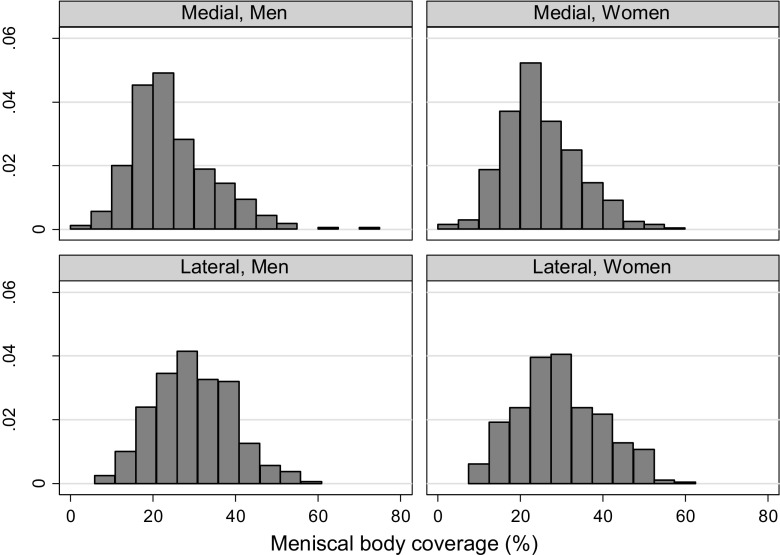
The distribution of the meniscal body coverage as percentage of the ipsilateral tibia plateau width in middle-aged and elderly persons from the general population, stratified by sex and compartment

The mean absolute meniscal coverage was 8.1 mm in the medial compartment and 9.2 in the lateral compartment, relatively similar in men and women. The mean coverage proportion (both sexes combined) was 28% in the medial compartment and 30% in the lateral compartment (Table 3, Figs. 2 and 3).

**Table 3:** Mean absolute meniscal coverage in mm and coverage proportion in middle-aged and elderly persons with Kellgren and Lawrence grade 0 knees

	Mean coverage, mm (SD)				Relative coverage, % (SD)*			
	Right knee		Left Knee		Right knee		Left knee	
	Medial	Lateral	Medial	Lateral	Medial	Lateral	Medial	Lateral
Men	7.8 (3.2)	9.9 (2.9)	9.9 (4.0)	10.0 (2.7)	24 (10)	30 (9)	31 (13)	31 (9)
Women	6.9 (2.5)	8.5 (2.9)	8.4 (3.4)	8.8 (2.5)	25 (9)	30 (10)	31 (12)	31 (10)
All	7.3 (2.9)	9.1 (3.0)	9.0 (3.8)	9.3 (2.6)	25 (9)	30 (10)	31 (12)	31 (9)

*Proportion (%) of ipsilateral tibial plateau width on mid-coronal MR image

### Evaluation of associations (cross-sectional) between the meniscal parameters and person characteristics

The multivariable analyses were made for medial and lateral compartment separately for the right knee. For the medial compartment, we found statistically significant associations with higher age (men only), and meniscal damage. No statistically significant associations were found for the lateral compartment (Table 4).

**Table 4:** The associations of meniscal body extrusion and meniscal body coverage with age, body mass index, and ipsilateral meniscal damage in middle-aged and elderly persons with Kellgren and Lawrence grade 0 in both knees, stratified by sex

	Medial compartment		Lateral compartment	
	Men	Women	Men	Women
Meniscal body extrusion (mm)*				
Age, per 10 years	*0.21 (0.05, 0.37)*	-0.03 (-0.17, 0.12)	0.04 (-0.11, 0.20)	-0.07 (-0.21, 0.07)
Body mass index, per unit	-0.01 (-0.04, 0.03)	-0.01 (-0.03, 0.01)	0.01 (-0.02, 0.05)	0.02 (-0.01, 0.04)
Ipsilateral meniscal damage	*1.02 (0.71, 1.34)*	*0.55 (0.20, 0.91)*	-0.01 (-0.31, 0.30)	0.14 (-0.20, 0.48)
Meniscal coverage proportion (%)				
Age, per 10 years	*-1.49 (-2.62, -0.36)*	-0.07 (-1.16, 1.01)	0.92 (-0.26, 2.09)	1.03 (-0.47, 2.53)
Body mass index, per unit	0.20 (-0.06, 0.45)	0.04 (-0.11, 0.19)	0.08 (-0.18, 0.34)	0.00 (-0.18, 0.19)
Ipsilateral meniscal damage	*-5.45 (-7.67, -3.23)*	*-4.63 (-7.24, -2.02)*	1.75 (-0.57, 4.07)	0.52 (-2.42, 3.46)

Presented numbers are mean differences in mm or % with 95% confidence intervals. Italicized font indicates statistically significant associations

*Model adjusted for tibia width

Data on extrusion and coverage proportion by gender and age can be downloaded as supplementary appendix (Web appendices 1 to 4). We only present data for the right knee. The results were similar also for the left knee.

## Discussion

In the literature, there is substantial lack of information on meniscal extrusion and coverage from persons *without* radiographic tibiofemoral knee OA. In our study, we found medial meniscus extrusion to be around 3 mm, but to be greater, and thus less cartilage coverage, if meniscus damage was present. High BMI per se was not found to be associated with meniscus extrusion and cartilage coverage.

Meniscus extrusion and loss/destruction of meniscus tissue are rather well-established features associated with knee OA [4–22, 31]. However, little has been done to determine these proxy measures of meniscus function to be expected in a general population free of tibiofemoral OA. This is needed to better understand was is “normal” and also potential gender differences have not been evaluated. At large, we found that women without OA had similar degree of meniscal body extrusion and cartilage coverage as men without OA. We further corroborate previous findings that the presence of ipsilateral meniscus damage was associated with meniscal extrusion in the medial compartment [8, 15, 17, 18, 23–25, 32–34]. Similar to prior reports, our cross-sectional data cannot answer the question whether meniscus damage, typically consisting of horizontal cleavages and/or flap tears (or in elderly women, simply meniscus maceration/destruction), is a result of a more mobile meniscus, or if the extrusion and displacement are a result of an already damaged meniscus and potentially disrupted hoop-tension. However, a prior longitudinal report based on Osteoarthritis Initiative data has provided evidence in support of the latter [25]. Either way, a damaged and extruded meniscus can no longer optimally fulfill its main purpose to distribute loads on the surrounding hyaline cartilage surfaces. As reported in earlier studies, meniscal body extrusion is a strong risk factor for cartilage loss [11, 12, 18, 19, 21, 32, 33, 35–38]. Such meniscal extrusion may be a strong causal intermediate in the chain of events leading to biomechanical joint failure and OA [39]. Thus, any risk factors that can potentially be modified or prevented, such as meniscus tears, are of key interest in order to reduce meniscus extrusion and the OA risk.

Our study had a number of limitations. As previously mentioned, since this is a cross-sectional study, one cannot make any causal inferences. Further, the mainly Caucasian population of Framingham limits generalizability of results. Still, there are, to the best of our knowledge, no similar data reported elsewhere from a randomly sampled general population. Further, we did not measure other parts of the meniscus than the meniscus body, thus, e.g., the position of the posterior horn needs further attention. This is because posterior partial or complete root tears often lead to meniscal body extrusion. We only used the mid-coronary slide for measurements. There are other more comprehensive methods [40], but ours is very easy to use and reproduce. The knee MRIs were taken in standard non-weight-bearing, and we cannot exclude the possibility of changes in meniscus position as compared to weight-bearing. However, currently, weight-bearing knee MRI is not available for epidemiologic studies or in the clinical setting. Also, the time of the day varied for the knee MRI examinations, and the effect on meniscus position during the daily cycle is unknown. Finally, we would like to comment on that a recent report on obese women suggested an association between knee symptoms and medial meniscal extrusion [24]. This is an interesting study question that warrants further attention, but then taking into account also other structural features on MRI indicative of knee OA such as synovitis and bone marrow lesions.

In conclusion, there is a need of epidemiologic data to better understand “normal” meniscal position on MRI in radiographically normal knees of both women and men. We found that the mean medial body extrusion in the general population of mainly Caucasian middle-aged and elderly persons without tibiofemoral OA is close to 3 mm, which corresponds to a commonly used cutoff in radiology to denote pathological extrusion [8]. This does not necessarily imply that the 3-mm cutoff is the most optimal with respect to sensitivity and specificity for other OA pathology for epidemiologic study purposes or longitudinal prediction of subsequent ipsicompartmental cartilage loss. Therefore, future aims will be to scrutinize the commonly used cutoff of 3 mm to denote pathological extrusion in association to radiographic OA and bone marrow lesions on MRI, as well as to study the association between knee symptoms and meniscal body extrusion. For the medial compartment, factors associated with meniscus position were predominantly ipsilateral meniscus tear or maceration/destruction. For the lateral compartment, no similar associations were found.

## Electronic supplementary material

ESM 1 (DOCX 19 kb)

## Abbreviations

BMI: Body mass index
CI: Confidence interval
ICC: Intra-class correlation coefficient
MR: Magnetic resonance
OA: Osteoarthritis
SD: Standard deviation
TW: Tibial width
MTPW: Medial tibia plateau width
LTPW: Lateral tibia plateau width
MMCW: Medial meniscal coronal width
LMCW: Lateral meniscal coronal width
MMCEx: Medial meniscal coronal extrusion
LMCEx: Lateral meniscal coronal extrusion
