# Supplementary material for: Meniscal body extrusion and cartilage coverage in middle-aged and elderly without radiographic knee osteoarthritis
Source: Eur Radiol. 2018 Oct 2;29(4):1848–54. doi: 10.1007/s00330-018-5741-3 (PMC6420611; doi:10.1007/s00330-018-5741-3)
Supplement: Supplementary file 1 — (DOCX 19 kb) [file 330_2018_5741_MOESM1_ESM.docx]

**Web appendix 1.** The mean (SD) medial meniscal body extrusion in mm in persons with Kellgren and Lawrence grade 0 knees.

| Sex | Age group (years) | Mean (SD) |
| --- | --- | --- |
| Men | 50-59 | 2.5 (1.1) |
| Men | 60-69 | 2.7 (1.4) |
| Men | 70-90 | 3.1 (1.4) |
| Women | 50-59 | 2.5 (1.0) |
| Women | 60-69 | 2.4 (1.0) |
| Women | 70-90 | 2.6 (1.3) |
| Sex | Body mass index | Mean (SD) |
| Men | <25.0 | 2.7 (1.3) |
| Men | 25.0-29.9 | 2.7 (1.3) |
| Men | 30+ | 2.6 (1.4) |
| Women | <25.0 | 2.5 (1.0) |
| Women | 25.0-29.9 | 2.6 (1.2) |
| Women | 30+ | 2.3 (1.0) |
| Sex | Meniscal damage | Mean (SD) |
| Men | No | 2.3 (1.1) |
| Men | Yes | 3.4 (1.5) |
| Women | No | 2.4 (1.0) |
| Women | Yes | 2.9 (1.4) |

**Web appendix 2.** The mean (SD) lateral meniscal body extrusion in mm in persons with Kellgren and Lawrence grade 0 knees.

| Sex | Age group (years) | Mean (SD) |
| --- | --- | --- |
| Men | 50-59 | 2.1 (1.2) |
| Men | 60-69 | 2.1 (1.3) |
| Men | 70-90 | 2.3 (1.3) |
| Women | 50-59 | 2.0 (1.2) |
| Women | 60-69 | 2.0 (1.2) |
| Women | 70-90 | 1.7 (1.0) |
| Sex | Body mass index | Mean (SD) |
| Men | <25.0 | 2.3 (1.2) |
| Men | 25.0-29.9 | 2.1 (1.2) |
| Men | 30+ | 2.2 (1.3) |
| Women | <25.0 | 1.9 (1.2) |
| Women | 25.0-29.9 | 1.8 (1.2) |
| Women | 30+ | 2.2 (1.3) |
| Sex | Meniscal damage | Mean (SD) |
| Men | No | 2.2 (1.2) |
| Men | Yes | 2.1 (1.3) |
| Women | No | 1.9 (1.2) |
| Women | Yes | 2.0 (1.3) |

**Web appendix 3.** The mean (SD) medial meniscal coverage proportion (%)* in persons with Kellgren and Lawrence grade 0 knees.

| Sex | Age group (years) | Mean (SD) |
| --- | --- | --- |
| Men | 50-59 | 26.0 (10.9) |
| Men | 60-69 | 23.9 (9.7) |
| Men | 70-90 | 21.4 (6.7) |
| Women | 50-59 | 25 (9.0) |
| Women | 60-69 | 24.9 (9.4) |
| Women | 70-90 | 24.2 (8.5) |
| Sex | Body mass index | Mean (SD) |
| Men | <25.0 | 22.2 (7.8) |
| Men | 25.0-29.9 | 24.9 (10.6) |
| Men | 30+ | 25.3 (10.1) |
| Women | <25.0 | 25.1 (8.8) |
| Women | 25.0-29.9 | 24.3 (9.5) |
| Women | 30+ | 25.4 (9.1) |
| Sex | Meniscal damage | Mean (SD) |
| Men | No | 26.3 (9.9) |
| Men | Yes | 20.5 (9.1) |
| Women | No | 25.7 (8.7) |
| Women | Yes | 20.8 (9.7) |

* Proportion (%) of medial tibial plateau width on mid coronal MR image

**Web appendix 4.** The mean (SD) lateral meniscal coverage proportion (%)* in persons with Kellgren and Lawrence grade 0 knees.

| Sex | Age group (years) | Mean (SD) |
| --- | --- | --- |
| Men | 50-59 | 29.3 (9.4) |
| Men | 60-69 | 30.2 (9.5) |
| Men | 70-90 | 30.9 (8.9) |
| Women | 50-59 | 29.2 (10.3) |
| Women | 60-69 | 29.9 (9.3) |
| Women | 70-90 | 30.9 (11.5) |
| Sex | Body mass index | Mean (SD) |
| Men | <25.0 | 29.0 (9.4) |
| Men | 25.0-29.9 | 30.3 (9.7) |
| Men | 30+ | 30.0 (9.0) |
| Women | <25.0 | 29.9 (9.3) |
| Women | 25.0-29.9 | 30.0 (10.6) |
| Women | 30+ | 28.8 (10.9) |
| Sex | Meniscal damage | Mean (SD) |
| Men | No | 29.4 (9.3) |
| Men | Yes | 31.0 (9.5) |
| Women | No | 29.4 (10.0) |
| Women | Yes | 30.7 (10.8) |

* Proportion (%) of lateral tibial plateau width on mid coronal MR image
